# Supplementary material for: Are carbon emissions trading and green financial instruments synergistic? -Comprehensive quantitative research based on content analysis
Source: PLoS One. 2024 Mar 7;19(3):e0298601. doi: 10.1371/journal.pone.0298601 (PMC10919601; doi:10.1371/journal.pone.0298601)
Supplement: S1 Data — (ZIP) [file pone.0298601.s001.zip › Data/地级市Z_F工作报告.zip!/╝¬┴╓/│ñ┤║╩╨/└┤╫╘╣1⁄2╓┌║┼í╛┬φ┐╦╩2╛▌═°í┐_2015.docx]

各位代表：

现在，我代表市政府，向大会作政府工作报告，请予审议，并请市政协委员提出意见。

一、2014年工作回顾

2014年是极不平凡的一年。面对世界经济复苏艰难、国内经济调速换挡的复杂严峻形势，全市上下在市委的正确领导下，从容应对挑战，奋力克难攻坚，全面推进改革开放和现代化建设，经济社会发展取得新的可喜成绩。

——经济平稳健康运行。预计地区生产总值增长7%。工业总产值突破1万亿元。粮食连续11年丰收。城镇新增就业目标超额完成。居民消费价格指数涨幅控制在2%以内。

——经济效益稳步提升。全口径财政收入增长7.3%。城市居民人均可支配收入增长9.8%。农村居民人均纯收入增长11%。规模以上工业企业利润增长19%。

——结构调整扎实推进。工业增加值率提高1.7个百分点。现代服务业增速超传统服务业4个百分点。科技成果转化率提升4个百分点。万元GDP能耗降低4.6%。

——改革攻坚顺利起步。行政审批、城市建设、农业农村、教育卫生、文化科技、安全生产、社会建设等重要领域和关键环节改革积极推进，成效已经开始显现。

——民生状况持续改善。第二个幸福长春行动计划全面实施，又为群众办了16类、49项民生实事。长春第七次获评“最具幸福感城市”。

过去一年，经济社会发展稳中有为、稳中提质、稳中有进，既有量的扩大，又有质的提升，更为今后奠定了基础。这将鼓舞全市人民砥砺奋进，不断创造新的业绩。

一年来，我们坚持稳中求进工作总基调，统筹稳增长、调结构、促改革、惠民生，采取了一系列既利当前、更惠长远的举措：

（一）积极克服下行压力，经济实现平稳健康增长。面对错综复杂的国内外经济形势和经济下行的巨大压力，我们坚持主动作为，充分挖掘增长潜力，努力激发市场活力，全力促进经济稳定增长。

统筹三次产业均衡增长。对产值超10亿元的重点工业企业和新增产值超亿元的重点项目跟踪包保，全方位帮扶重点困难企业，确保工业持续增长。及时取消限购政策，稳定住房消费。支持自主品牌汽车消费，积极扩大旅游、信息、文化、养老服务消费。繁荣市场，搞活流通，社会消费品零售总额增长12%。努力克服春季低温、局部伏旱影响，粮食产量达到230亿斤左右。

扩大投资规模拉动增长。增加财政预算内投资，重点投向地铁、“两横三纵”快速路、保障性安居工程等领域。采取股权投资、贷款贴息等方式，引导社会资本投资战略性新兴产业、现代服务业，充分发挥政府投资“四两拨千斤”的带动作用。深化投融资体制改革，落实企业投资自主权，民间投资增长20%。在经济环境趋紧、企业投资意愿不足的情况下，全市固定资产投资以15%的速度快速增长。

释放市场活力刺激增长。全面实施简政放权，行政审批由823项减少到251项，市本级非行政许可实现“零审批”，切实减少政府对市场的直接干预。推动工商登记制度改革，新注册市场主体8.5万户，新登记私营企业接近2万户，形成了新的创业促就业热潮。11.1万户小微企业享受结构性减税政策，3.4万户企业纳入“营改增”试点，减轻企业税负7.3亿元。这些措施放开了市场、帮扶了企业，直接增强了经济增长动力。

（二）坚定不移调整结构，转型升级取得明显成效。着眼阻碍发展的结构性问题，我们坚持立足实际、突出重点，在稳定增长中调结构、增效益，在创新驱动中抓转型、促升级，为长远发展铺路搭桥。

积极优化工业结构。实施奥迪Q系列30万辆整车、大成异山梨醇、长客装备搬迁改造等630个投资超亿元项目，三大支柱产业稳步壮大。新能源汽车产能达到1.5万辆，聚乳酸产业异军突起，新材料、生物化工、医药健康、光电信息等产业园区聚集效应初步形成。工业投资增长18%，五大战略性新兴产业投资增长23%。

全面优化农业结构。新建万亩粮油高产示范片120个、标准化养殖示范区200个。农机化综合作业水平提升到76.5%，玉米、水稻机收率分别达到50.1%和80%。农民合作社、家庭农场数量增加一倍以上。土地流转面积达到总耕地面积24.6%。增加科学储粮仓2.5万套。“长春松花江大米”品牌推介取得明显成效。

大力优化服务业结构。实施投资5亿元以上现代服务业项目46个。商务综合体累计开工面积500万平方米。文化创意、信息服务等十大现代服务业集聚区入驻企业突破2000户。玉米、汽车两大电商平台营业额均超过200亿元。物流快递、移动互联网等新兴业态竞相成长。引进域外金融机构3家。2户企业上市，5户企业“新三板”挂牌。

稳步优化区域结构。制定出台了生产力布局优化调整意见，促进开发区、城区、县域协调发展。积极引导开发区转型升级，加快发展方式转变。提高主城区周边土地投资强度和节约集约利用水平，推动制造业向县域有序梯度转移，县域经济加速崛起。积极稳妥地推进新型城镇化，6个示范镇建设取得初步成效。九台正式撤市设区。

推进创新驱动发展。深入开展产学研协同创新机制试点，新增高新技术企业33户、科技“小巨人”企业68户。加快北湖科技园等创新平台建设，新增科技企业孵化器60万平方米。成功突破120项关键技术，“吉林一号”卫星工程实现重大突破。科技金融创新服务中心挂牌运营，促进了科技与金融的深层次融合。

（三）全面实施各项改革，经济发展活力明显增强。主动适应经济发展新常态，坚持把深化改革作为关键举措，统筹谋划、分类推进，努力向改革要动力、要潜力、要红利。

积极开展改革创新试点。新型城镇化、老工业区搬迁改造、服务业综合改革、生物制造区域集聚发展、生物基材料制品应用、水生态文明建设等多项工作被列为国家级改革创新试点，不仅得到了政策资金支持，更通过先行先试争取了主动。

全力实施重点领域改革。民营经济综合配套改革示范区试点全面铺开，并取得显著成效。国有企业改革取得新进展。行政事业单位国有资产管理改革扎实推进。向城区下放城市管理权限。农村土地收益保证贷款试点稳步实施。安全生产监管体制改革实现突破。

扎实推进政府自身改革。完善食品药品监管体制，调整工商、质监管理体制，整合卫生、计生等相关机构，新一轮政府机构改革基本完成。撤销89个议事协调机构。事业单位分类改革基本完成。市本级公共财政收支预算、市直部门“三公”经费预算全部公开。

利用对外开放促进改革。全面强化招商引资，实际利用外资、内资分别增长12%和15%。兴隆综合保税区封关运营，跨境电子商务业务全面启动。轨道客车出口订单大幅增加，机电、高新技术产品占出口总额比重稳步提高。接待国内外游客人数增长15%。长春至香港直飞航线通航。

（四）加快建设新型城市，城市宜居水平稳步提升。面对城市化进程不断加快带来的挑战，坚持把绿色、智慧、人文理念贯穿到城市建设管理全过程，多措并举、综合施策，推动城市在承载能力扩大的基础上不断提高综合质量。

构建立体交通体系。历时两年零八个月、经过4万余名建设者昼夜奋战，全长84.5公里的“两横三纵”快速路全线投入运行。这标志着长春正式跨入立体交通时代，意味着我们的城市有了更快的节奏、更高的效率，也见证了长春人民的速度和激情、干劲和魄力。机场快速路等重点工程完成序时进度。大中修道路90条。地铁1号线、2号线建设稳步实施。北湖快轨启动建设。55路有轨电车正式开通。城市交通指挥管理系统开始实施升级改造。

强化市政公用服务。五水厂投入运行，一、三水厂改扩建完成主体工程。新建改造二次供水泵站241座、二次供水管网1600公里。新增供热能力1460万平方米。改造燃气高危管网150公里、供热管网210公里。29.2万农村人口饮水安全问题得到解决。

防治大气环境污染。淘汰燃煤小锅炉1178台。注销黄标车、老旧机动车1.45万辆。强力实施燃煤发电机组脱硫、脱硝、除尘改造。从严整治工业企业烟粉尘排放和建筑工地扬尘。PM10、PM2.5浓度均值分别下降8.5%和6.8%，空气质量优良天数有所增加。

综合整治市容环境。集中治理火车站周边、长吉南线出入口等区域秩序，努力消除城市管理顽疾。新建续建公园13个，新建大块绿地39块，补植街路143条，新增城市绿地600公顷。植树造林5818公顷。清收非法占用林地30758公顷。

提升城市文化品位。加强城市规划管理。强化历史文化遗存保护。长影博物馆建成开放。群众艺术馆、朝鲜族艺术馆竣工。雕塑公园被评为“创造未来文化遗产”示范单位。评选推出长春旅游“新十五景”。巩固提升国家公共文化服务体系示范区创建成果。毫不松懈地开展文明城市创建活动。“智慧长春”建设全面展开。

（五）全力以赴改善民生，努力提高群众的幸福感。牢固树立“民生是发展之本、稳定是和谐之基”理念，坚持把改善民生作为政府全部工作的出发点和落脚点，竭诚尽力为群众办好事、办实事。

提高群众收入水平。积极扶持高校毕业生、城镇就业困难人员就业。开发就业岗位14.6万个，新增就业12.7万人。转移农村剩余劳动力110.6万人次。妥善解决6.3万名被征地农民参保问题。城镇职工养老保险新增参保人员11万人。失业保险金提高181元。企业退休人员养老金人均提高159元。城乡居民收入增长快于经济增长，农民收入增长快于城镇居民收入增长。

帮扶救助困难群众。城乡居民最低生活保障标准提高8%，五保对象供养标准提高23%。城区低保家庭老、少、病、残人员补助上浮50%。制定出台城乡居民临时救助办法，妥善解决群众突发性、临时性生活困难。提高残疾人教育、康复项目补助标准，近万名残疾人受益。城区困难群众患急危重症到定点医疗机构就医实现“先住院、后付费”。困难群众法律援助基本做到应援尽援。

改善居民居住条件。新建廉租房600套、公租房1700套。拆除棚户区203万平方米，建设回迁房1.83万套。46个老旧散住宅区完成改造。90%以上的老旧散住宅区实现多种形式物业管理。扎实推进幸福社区建设，40%以上的城市社区服务用房达到1000平方米。绿化美化村屯70个。100个村屯农村人居环境综合整治达标。

完善便民利民服务。开辟优化公交线路13条，新增更新公交车辆453台。改造居民巷道1194条。新建停车泊位2371个。新建农村公路1286公里，改造危桥129座。全面加大对养老机构政策资金扶持力度，新建养老机构39个，增加养老床位4419张。二级以上医院对养老机构实行对口医疗服务。开展医疗保险晚期癌症患者临终关怀服务。

强化基本公共服务。新建改建15所公办幼儿园。新建4所九年制学校。城区学校常规教学、信息技术装备全部达标。创建30所新优质学校，2所“公办民助”学校退回公办，754名教师交流轮岗，教育均衡水平稳步提升。圆满有序完成中高考。居民大额补充医疗保险病种扩大到所有疾病，22种大病实现低自付治疗。城区新增健身步道10条、健身路径100套。我市运动员在索契冬奥会、南京青奥会、仁川亚运会获得好成绩。

加强社会综合治理。积极构建“人性化、网格化、信息化、法治化”社会治理模式，深入创建平安社区、平安校园、平安家庭，群众安全感稳步提升。加强社会治安防控体系建设，新增治安高清探头1万余个，110刑事警情、“两抢”案件数量分别下降19.2%和14.4%，命案等有重大影响案件全部告破。累计排查整改安全生产隐患6.4万项，关停查封企业614家，安全生产形势稳步好转。创建食品安全城市，食品安全事故发生起数下降30.8%。

（六）积极推进依法行政，政府自身建设得到加强。巩固拓展党的群众路线教育实践活动成果，坚持重法治、转作风、促和谐，全面增强政府执行力和公信力。

主动接受监督。自觉接受人大及其常委会的法律监督、工作监督和政协的民主监督，坚持重大事项向人大报告、与政协协商制度。认真听取各民主党派、工商联以及无党派人士的意见建议。办理人大议案6件、人大代表建议189件、政协建议案2件、政协提案279件。

加强依法行政。全面强化对行政权力的制约和监督，严格规范公正文明执法。改革行政复议体制。强化行政监察。加大涉软案件查处力度。

切实转变作风。全面贯彻中央八项规定精神。认真开展第二批党的群众路线教育实践活动。切实加强政府廉政建设和反腐败工作，一批违法违纪分子受到惩处。

努力化解社会矛盾。拓宽信访接待渠道，提高市长公开电话、局长接待日、读报读网工作水平，一批重大信访疑难案件结案息访。

深入开展双拥创建工作。隆重举行烈士纪念日、抗战胜利纪念日活动。国家安全、司法行政、审计、质监、统计、民族、宗教、外事、侨务、会展、供销、气象、地震、档案、人防、公积金、地方志、红十字、妇女儿童等工作都取得了新成绩。

各位代表，过去一年发展取得的成绩告诉我们，只有紧紧依靠党中央、国务院，省委、省政府和市委的正确领导，我们才能把握方向、坚定信心；只有紧紧依靠市人大、市政协的监督支持，我们才能汇集智慧、形成合力；只有紧紧依靠全市上下的团结奋斗，我们才能勇往直前、勇夺胜利。在此，我代表市政府，向全市广大干部群众致以崇高的敬意！向中省直驻长单位、人民解放军和武警驻长部队以及海内外的朋友们，表示衷心的感谢！

成绩来之不易，问题不容忽视。在为取得的成绩欢欣鼓舞的同时，我们也必须清醒地认识到，前进道路上还有不少困难和问题。主要表现在：经济下行压力较大，转型升级带来的阵痛开始显现；创新驱动机制尚不完善，经济增长的内生动力有待增强；空气污染等问题日益显性化，节能减排任务艰巨；就业结构性矛盾突出，住房、医疗、养老、教育、收入分配、征地拆迁等方面群众不满意的问题依然较多；安全基础设施不完善，安全事故高发势头还没有从根本上得到遏制；政府依法行政水平不高，公职人员不廉不勤现象仍然存在。对此，我们将高度重视，并在今后工作中认真加以克服和解决。

二、2015年工作安排

2015年，是全面深化改革的关键之年，是全面推进依法治市的开局之年，也是全面完成“十二五”规划的收官之年。尤其要充分认识到，当前乃至今后一个时期，我们将长期面临经济发展新常态。从经济上看，增速换挡、动力转换，给我们组织经济建设、加快发展转型带来新的压力；从社会上看，利益格局深刻调整、新旧矛盾交织叠加，给我们维护稳定、促进和谐带来新的挑战；从政治上看，推进依法治市、加强作风建设，给我们依法行政、科学行政提出新的要求。新常态也是新机遇，新常态要有新作为。必须看到：长春正处于重要的发展战略机遇期，经济韧性好、潜力足、回旋空间大，长期向好的基本面没有变，我们有坚实的发展基础；我们已经制定并实施了一系列措施，努力应对新挑战、抓住新机遇、培育新动力，积极效应正在逐步显现，我们有充分的发展准备；特别是中央振兴东北、省里支持长春的力度不断加大，广大干部群众激情满怀、干劲十足，我们有坚强的发展后盾。只要坚定不移地在省委、省政府和市委的正确领导下，积极适应，主动引领，全力抢抓新机遇、奋力迎接新挑战，就一定能够推动长春在经济新常态下实现新发展、开创新局面。

政府工作的总体思路是：全面落实党的十八大和十八届三中、四中全会精神，深入学习贯彻习近平总书记系列重要讲话精神，按照省委十届四次全会、市委十二届六次全会要求部署，牢牢抓住国家新一轮振兴东北的重大机遇，紧紧围绕“科学发展、加快振兴，努力让城乡居民生活得更加美好”总目标，突出发挥“五个优势”、推进“五项举措”、加快“五大发展”，突出中部创新转型核心区建设任务，集中精力抓发展、抓改革、抓民生、抓稳定、抓落实，全面推进依法治市，全面推进城市转型升级，加快建设幸福长春。

全市经济社会发展的主要预期目标是：地区生产总值增长7.5%左右，财政收入与经济保持同步增长，万元GDP能耗降低2%，城市居民人均可支配收入增长10%左右，农村居民人均纯收入增长10%左右，城镇登记失业率控制在4%以内，居民消费价格指数涨幅控制在3%左右。

今年政府各项工作要牢牢把握稳中求进的总基调。“稳”的重点是稳住经济运行，确保增长、就业、物价不出现大的波动。“进”的重点是调整经济结构和深化改革开放，确保转变发展方式和改善民生工作取得新成效。我们要统筹好“稳”和“进”的关系，做到两不误、实现双胜利。

（一）努力保持经济稳定增长

稳增长仍然是今年政府工作的突出任务。我们要充分挖掘各种增长潜力，确保经济运行在合理区间，为群众提供充足的就业机会，为市场提供稳定的心理预期，为转型升级提供有力支撑。

发挥投资的关键作用。建立健全政府和社会资本合作（PPP）模式，积极推动社会资本参与基础设施建设运营。创新重点领域投融资机制，打破市场壁垒，降低准入门槛，鼓励和促进民间投资。支持企业上市，扩大直接融资规模。全年固定资产投资力争达到4500亿元以上。

发挥消费的基础作用。培育文化、旅游、健康、养老、信息等大众服务性消费。稳定住房消费，保持房地产市场平稳健康发展。推进物流共同配送，降低流通成本。多渠道增加居民收入，落实消费政策，强化消费维权，努力让群众能消费、愿消费、敢消费。社会消费品零售总额增长12%左右。

发挥出口的促进作用。支持企业设立境外产品展示营销中心，鼓励轨道客车、汽车零部件等优势产品出口。大力发展口岸经济。加快通关便利化改革。用好72小时过境免签政策。完善兴隆综合保税区功能，积极培育保税物流、服务外包、跨境电子商务产业。

（二）加快推动创新城市建设

创新是城市兴旺发达的不竭动力。我们要采取更加有力的措施，激发、释放社会的创造力，发现、培育新的增长点，努力营造大众创业、万众创新的生动局面。

突出科技创新。充分发挥在长科研院所、大专院校作用，加快完善产学研协同创新机制，实施100项重大技术攻关，转化100项重点科技成果，培育100户科技“小巨人”企业。支持企业引进新技术、开发新产品，促进传统产业转型升级。强化企业创新主体作用。

实施产业创新。推动工业化和信息化深度融合，促进生产性服务业与先进制造业良性互动。通过设立投资基金等多种形式，引导民间资本开发新产业、新业态、新商业模式。积极适应消费个性化、多样化趋势，促进工业生产小型化、专业化、智能化。

强化环境创新。加快实施民营经济综合配套改革示范区试点，努力破除各种体制机制束缚，最大限度释放微观主体活力。落实结构性减税政策，加强创业金融服务，加大对新兴产业、小微企业支持力度，努力让一切想创新、能创新的人有机会、有舞台。

（三）着力调整优化经济结构

调结构是转型升级的必由之路。我们要狠抓增量投入，促进传统产业向中高端迈进，增强战略性新兴产业和服务业的支撑作用，推动发展方式向质量效率型集约增长转变。

大力发展先进制造业。加快奥迪Q系列30万辆整车项目建设，支持一汽通用引进新车型，扩大中高端整车产能。与一汽、长客等央企共建产业园区，突出发展核心零部件，壮大产业集群。支持玉米化工重点企业战略合作重组，延伸产业链条。全年实施投资3000万元以上项目800个，完成投资增长16%以上。

培育战略性新兴产业。充分发挥国家级开发区创新型园区载体作用，实施聚乳酸、激光设备、智能机器人等100个投资超亿元项目，加快发展医药健康、生物制造、光电信息、新能源、新材料产业，努力抢占未来发展制高点。战略性新兴产业投资增长20%以上。

优先发展现代服务业。积极开展服务业综合改革试点，着力发展现代物流、电子商务、文化创意、移动互联网等现代服务业。大力发展会展业。做大做强旅游业。加快建设商务综合体。着力引进金融机构，培育现代保险、金融大数据、互联网金融、金融后援服务业，建设东北亚区域性金融服务中心。现代服务业投资增长15%。

加快发展现代农业。稳定粮食生产。积极引导农民瞄准市场，调整种养结构，提高农产品质量效益。玉米保护性耕作面积达到240万亩。农机化综合作业水平达到78%左右。土地流转面积达到总耕地面积27%左右。继续推介“长春松花江大米”品牌。实施畜牧业“全产业链”建设、无规定动物疫病区建设。

（四）优化经济发展空间布局

现在，长春已经形成了多层次的发展空间。我们要突出特色、分类指导、统筹兼顾，促进各发展空间优势互补、错位竞争、协调发展。

城区要成为服务业的主战场。大力发展商务楼宇、特色街区和服务业集聚区，尽快使相对集聚成为服务业发展的重要模式。全年实施投资超亿元项目300个以上。加快铁北老工业区搬迁改造。支持双阳、九台加快发展，尽快成为现代化新城区。

开发区要成为转型升级的主力军。集中精力发展新型工业化，强化创新驱动，培育产业集群，加快转型升级。开发区工业投资要力争增长20%。支持国家级开发区发挥带动作用，与县域省级开发区合作建设新区。

县域要成为产城融合的主阵地。高标准建设县城，发展社会事业，吸引农村人口进城。加快建设省级工业开发区，壮大产业规模，促进农村劳动力转移。积极推动6个示范镇开展新型城镇化试点，莲花山度假区尽快实现全域城镇化。启动村庄规划编制，建设美丽乡村。

长春要在吉林中部创新转型核心区建设中发挥龙头带动作用。加快长吉一体化进程，着力构建哈长城市群，推动长吉图战略实施。逐步畅通与公主岭、伊通交通联系，强化中心城市集聚扩散能力，通过辐射周边、服务全省发展自己。

（五）全面推进重要领域改革

坚持把改革作为激发活力、增添动力的根本举措。我们要推出既具有年度特点、又有利于长远制度安排的改革措施，更多释放改革红利。

加快政府自身创新带动改革。建立政府权力清单、责任清单、负面清单，进一步简政放权。改进行政审批服务，大幅减少前置审批，优化流程、提高效率。政府预算、决算、“三公”经费都要向社会公开，让群众看明白、能监督。规范政府举债融资机制，把政府性债务纳入预算管理，积极防范化解债务风险。

坚持围绕问题导向推进改革。积极推进不动产统一登记。完善国资国企监管体制，发展混合所有制经济。加快社会信用体系建设。推进教育、医疗、文化、体育领域改革，不断提高公共服务均等化水平。稳步实施安全生产、食品安全、群众信访等方面改革，促进社会和谐。

推动新一轮高水平对外开放。加大招商引资力度，引进国际战略投资者、央企、民营500强企业，实际利用内外资分别增长15%和13%。加快建设中德、中韩、中白、中俄等国际合作产业园区，争创国家级承接产业转移示范区。

（六）提高城市建设管理水平

建设幸福长春，必须首先建好幸福城市，让群众生活得更美好。我们要坚持以人为本、建管并重、生态优先，全力建设绿色、智慧、人文的新型城市。

加快完善立体交通体系。完成“两横三纵”快速路辅助工程。完善南部新城、城市东部等区域性路网。续建地铁1、2号线和北湖快轨。打通一批断头路、卡脖路。建设一批人行过街天桥。鼓励社会力量建设停车场。交通指挥管理系统改造升级一期工程投入使用。

扎实提升城市承载能力。一、三水厂改扩建工程竣工投入运行。新增供热能力500万平方米。改造燃气高危管网150公里、供热管网200公里。天然气外环高压管网、调峰储气设施建成投入使用，加强气源调度保障，缓解冬季供应紧张。推进智能电网建设，提高供电保障能力。积极承接一汽水电气热和基础设施服务保障功能。

稳步推进“智慧城市”建设。提高宽带家庭覆盖面，力争公共热点区域免费无线网络（WiFi）基本实现全覆盖。统筹推进智慧政务、智慧城管、智慧交通、市民一卡通等信息化应用工程，提升城市运行智能化水平。

争创“公交都市”示范城市。新增更新公交车辆，调整优化公交线路10条以上。规划建设一批公交换乘节点、首末站、港湾式停靠站、公交专用车道，延长公交服务时间。稳步推进公交体制改革，加强行业管理，提高服务水平。

创建国家历史文化名城。加强重点区域、关键节点的城市设计，保护修复历史文化街区、历史建筑。启动建设雕塑艺术博物馆。改造市方志馆。市博物馆、美术馆、规划展览馆正式开放。培育践行社会主义核心价值观。创建文明城市，提高市民文明素质。推动军地军民融合发展，争创全国双拥模范城“八连冠”。

努力提升城市管理水平。扎实开展市容环境综合整治。治理城市出入口环境。打造一批精品街路。下大气力清除和防治违法建筑。今年，我们要把主要精力从城市建设转到城市管理上来，努力实现城市管理精细化、长效化，让市容环境更干净、更整洁、更有序。

（七）切实加强生态环境保护

生态环境关系人民幸福，关乎城市可持续发展。我们要把生态环境保护工作摆上更加突出的位置，下决心用硬措施完成硬任务。

强化污染防治。四环以内，严禁燃放烟花爆竹，渣土、垃圾必须密封运输，建筑工地必须落实扬尘防控措施。下大气力淘汰黄标车、老旧机动车和10吨以下燃煤小锅炉。串湖、北郊污水处理新建扩建工程完工并投入运行。全面落实“禁塑令”。

强化节能减排。积极调整能源结构。强化煤炭清洁高效利用。实施资源性产品价格改革，居民用水、用气实行阶梯价格。加快推广应用新能源汽车。推行农业标准化清洁生产。发展节能环保产业。

强化生态建设。新建续建净月生态文化园等14个公园。完成“两横三纵”快速路沿线绿化。实施四环路绿化改造。植树造林3000公顷。非法占用林地基本完成清收，并尽快还林。积极创建国家森林城市。加强伊通河综合整治。实施莲花山还湿还林还水工程。积极开展水生态文明建设试点。

（八）全力提升群众幸福指数

人民群众对幸福生活的向往，就是我们的奋斗目标。我们要制定实施第三个幸福长春行动计划，坚持不懈地为群众办好事、做实事。

守住基本生活底线。实施更加积极的就业政策，努力克服经济调速影响，确保就业形势基本稳定。加大对高校毕业生、城镇就业困难人员就业帮扶力度，“零就业家庭”保持动态为零。提高城乡居民基础养老金标准。探索推行失能人员照护保险制度。完善失业、工伤保险制度。不断完善以“保基本、救急难”为核心的大救助体系，全力保障困难群众基本生活。加强残疾人基本公共服务，支持慈善事业，让每一个身处困境者都能得到社会关爱和温暖。

补长公共服务短板。对普惠性幼儿园给予经费奖补。深化大学区改革，强化学校设施标准化建设，不断提高基础教育质量。100%小学、90%初中实现免试就近划片入学。大力发展现代职业教育。城区健身路径基本实现社区全覆盖，乡镇和60%以上行政村配备健身器材。加快村级卫生所标准化建设。实施健康长春行动计划，倡导健康生活方式，提升市民健康水平。完善公共文化服务体系，繁荣群众文化生活。积极推动流动人口公共服务均等化。加快幸福社区建设。

突破热点难点问题。改造100万平方米棚户区和危旧房屋、130万平方米“老旧散弃”住宅区、650万平方米“暖房子”。新建2000套公租房，5000套保障房年内完成配租。突出解决物业小区弃管问题。新建改造二次供水泵站370座，力争再用一年时间基本完成主城区二次供水设施改造。鼓励社会力量建设养老机构。加快城区托老中心、农村居家养老服务大院建设。新建农村公路1000公里，不断改善农村出行条件。

（九）强力维护城市安全稳定

发展要安全，安全才能发展。我们要始终牢记“生命至上、安全第一”，时刻绷紧安全这根弦，用最严格的监管、最严厉的处罚、最严肃的问责，维护社会平安、和谐、稳定。

全面强化安全生产。完善安全生产防控体系，加大安全监管力度，坚决做到减少一般事故、防范较大事故、遏制重大事故、杜绝特大事故，创建国家安全发展示范城市。

切实强化社会治理。创新社会治安防控体系，严厉打击各类刑事犯罪，坚持命案必破、侵财必打、逃犯必追、黄赌毒必治、黑恶势力必除，进一步增强群众安全感。

不断强化食品安全。健全食品药品安全监管体系，以“零容忍”的态度严惩食品药品违法行为，坚决治理餐桌上的“污染”，切实保障“舌尖上的安全”。

着力强化公共安全。突出消防、交通等重点领域安全整治，降低安全事故伤亡人数。推进城市安全设施建设，加强应急培训演练。创建国家防震减灾示范城市。

突出强化信访接待。畅通民意诉求渠道，调处纠纷、化解矛盾、促进和谐。完善落实重大信访案件包保制度，切实解决信访群众合理合法诉求。

（十）积极推进依法治市进程

治市必先治政。我们要深入贯彻依法治国基本方略，认真落实市委关于全面推进依法治市的实施意见，以建设法治政府为突破口，统筹做好政府立法、执法、普法等各领域工作，奋力开创依法治市的新局面。

依法履行政府职能。推动机构、职能、权限、程序、责任法定化，切实做到法无授权不可为、法定职责必须为，坚决纠正不作为、乱作为，坚决克服懒政、怠政，坚决惩处失职、渎职。

推进法治领域改革。健全依法决策机制，创新政府立法机制，改革行政执法体制，坚持严格规范公正文明执法，强化对行政权力的制约和监督，努力把政府工作全面纳入法治轨道。

加快建设法治社会。积极开展普法宣传教育。围绕征地拆迁、企业用工、物业管理等十个热点难点问题强化公共法律服务。健全依法维权和化解纠纷机制，保证群众合法权益得到有效保护。

各位代表！做好政府工作，必须加强自身建设。

我们要自觉接受市人大及其常委会的法律监督和工作监督，接受市政协的民主监督，主动听取各民主党派、工商联以及无党派人士的意见建议。

我们要厉行节约、反对浪费，坚持过紧日子。严格执行“约法三章”：政府机关楼堂馆所一律不得新建，财政供养人员总量只减不增，“三公”经费只减不增。

我们要巩固和拓展党的群众路线教育实践活动成果，以锲而不舍的决心和毅力，把作风建设不断引向深入。所有公务员都要以人民利益至上，廉洁奉公，勤勉尽责，当好人民公仆。

各位代表！人民的期待殷切而又厚重，肩负的使命光荣而又艰巨。让我们在市委的正确领导下，抢抓机遇，真抓实干，奋力拼搏，为加快城市转型升级、建设幸福长春而努力奋斗！
